# Optogenetic activation of the ventral tegmental area-hippocampal pathway facilitates rapid adaptation to changes in spatial goals

**DOI:** 10.1016/j.isci.2023.108536

**Published:** 2023-11-23

**Authors:** Yuta Tamatsu, Hirotsugu Azechi, Riku Takahashi, Fumiya Sawatani, Kaoru Ide, Fumino Fujiyama, Susumu Takahashi

**Affiliations:** 1 Laboratory of Cognitive and Behavioral Neuroscience, Graduate School of Brain Science, Doshisha University, Kyotanabe 610-0394, Japan; 2 Laboratory of Histology and Cytology, Faculty of Medicine, and Graduate School of Medicine, Hokkaido University, Sapporo 060-8638, Japan

**Keywords:** Biological sciences, neuroscience, cognitive neuroscience

## Abstract

Animal adaptation to environmental goals to pursue rewards is modulated by dopamine. However, the role of dopamine in the hippocampus, involved in spatial navigation, remains unclear. Here, we studied dopaminergic inputs from the ventral tegmental area (VTA) to the hippocampus, focusing on spatial goal persistence and adaptation. Mice with VTA dopaminergic lesions struggled to locate and update learned reward locations in a circular maze with dynamic reward locations, emphasizing the importance of VTA dopaminergic neurons in the persistence and adaptation of spatial memory. Further, these deficits were accompanied by motor impairments or motivational loss even when dopamine receptors in the dorsal hippocampus were selectively blocked. Stimulation of VTA dopaminergic axons within the dorsal hippocampus enhanced the mice’s ability to adapt to changing reward locations. These findings provide insights into the contribution of dopaminergic inputs within the hippocampus to spatial goal adaptation.

## Highlights

• VTA-hippocampal dopaminergic pathway enables quick spatial goal shifts
• Impaired VTA dopaminergic neurons disrupt spatial reward memory recall
• Optogenetic stimulation of the pathway enhances adaptation to new reward locations

## Graphical abstract

**Figure:**
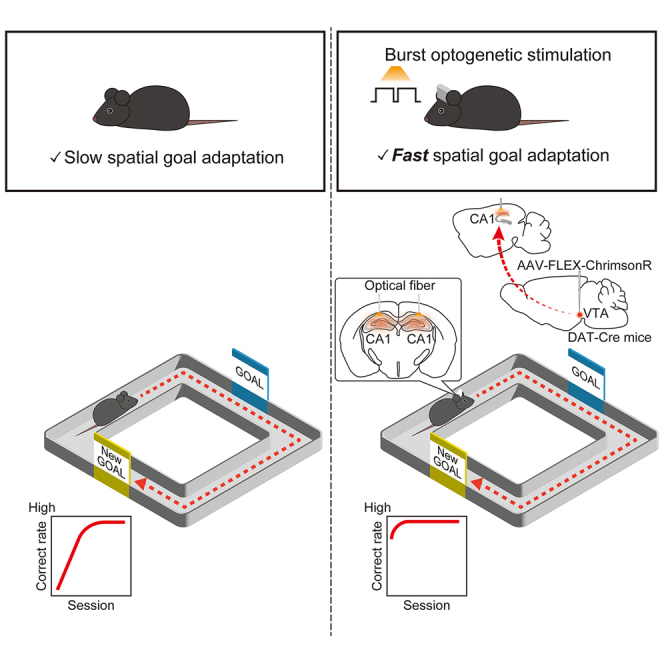

## Introduction

Goal-directed spatial navigation is a fundamental aspect of animal behavior that enables the pursuit of essential resources such as food, shelter, and mates. Rewards serve as a driving force in such navigational behaviors, with agents utilizing reinforcement learning to adapt their behavior to maximize rewards in their environment.^1^ Multiple lines of evidence indicate that the neuromodulator dopamine functions to generate a reward-related signal for the basal ganglia circuitry in the brains of animals, which is consistent with the reinforcement learning theory.^2^^,^^3^^,^^4^^,^^5^ However, the specific role of dopamine in the hippocampus, a brain structure deeply involved in spatial navigation and in encoding of an animal’s current location by place cells, remains unknown.

With the identification of the ventral tegmental area (VTA) and locus coeruleus (LC) as sources of dopaminergic inputs to the hippocampus, research has demonstrated that the VTA and LC contribute to the preservation of learned behavioral performance in the hippocampus.^6^^,^^7^^,^^8^^,^^9^^,^^10^ Recent investigations have enriched our understanding of hippocampal function by discovering reward cells in the dorsal hippocampus that concurrently encode the reward location and the animal’s position.^11^ The interaction between dopaminergic inputs and reward cell activity remains unclear, although the firing patterns of these reward cells display robust associations with goal locations. Furthermore, previous pharmacological studies showed that dopamine modulates the response to alterations in goal locations during goal-directed navigational behaviors.^12^^,^^13^^,^^14^ Consequently, the aim of this study was to examine the impact of the VTA dopaminergic input on the hippocampus in the context of goal adaptation during spatial navigation. Our study provides valuable insights into the role of dopaminergic inputs in the dorsal hippocampus in facilitating the rapid adaptation of goal locations during spatial navigation.

## Results

### Learning of fixed and changing reward location tasks

**Figure 1:**
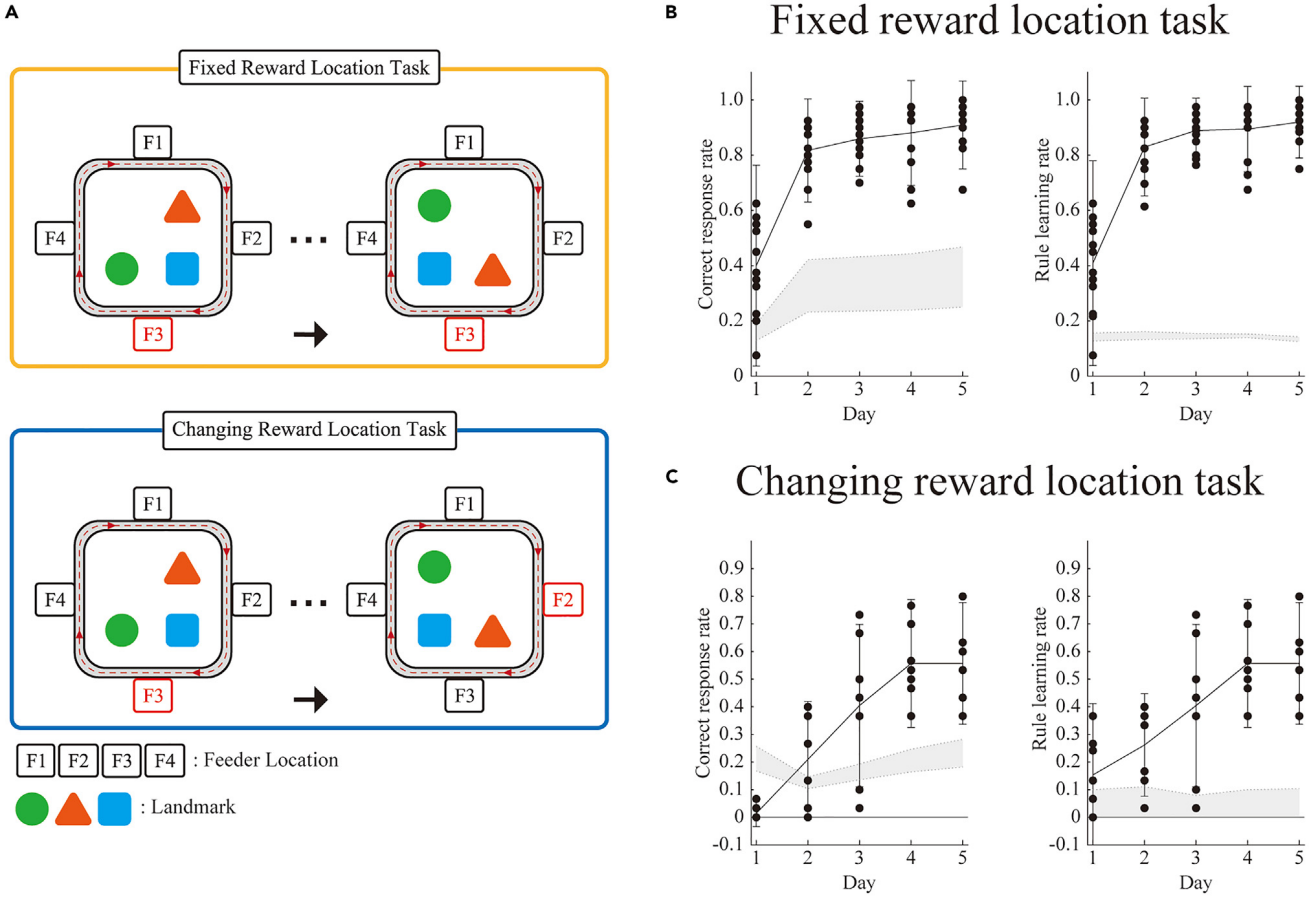
Behavioral paradigm and maze configuration for spatial learning tasks in mice (A) Maze schematics. Top: Example configuration for the fixed reward location (FRL) task, with the rewarded feeder (F3) remaining in a fixed location after each randomized rotation of the landmark objects. Bottom: Example configuration for the changing reward location (CRL) task, in which the rewarded feeder (F3) is relocated after each randomized rotation of the landmark objects. (B) Correct response rate (left) and rule learning rate (right) during the FRL task performance. The gray shaded area represents the shuffled distribution of both rates, which exceed the chance level from day 2 onward. (C) Correct response rate (left) and rule learning rate (right) during the CRL task performance. The gray shaded area represents the shuffled distribution of both rates, which exceed the chance level from day 4 onward. The dots represent raw data points for individual mice, while the graph indicates mean ± standard deviation (SD).

In this study, we examined the behavioral responses of mice in a circular maze, particularly focusing on their ability to adapt to fixed and changing reward locations. The experiment was conducted in a soundproof room, with the maze designed to have four possible reward locations: east, west, south, and north. The reward was food pellets dispensed from a single dispenser. We designed two tasks, a fixed reward location task and a changing reward location task, to assess the adaptation of mice to the changing reward locations. In both tasks, the mice were trained to locate the reward based solely on distal objects in the soundproof room. The researchers achieved this by interfering with the proximal landmark cues, as the three prominent objects in the maze were randomly rearranged from session to session (Figure 1A).

In the fixed reward location task, the mice were trained to locate the reward at a fixed location. We calculated the correct response rate, defined as the number of laps in which the animal only chose a reward-providing feeder, divided by the total number of laps. The mice achieved an average correct response rate of 80% on the second day of training, with all mice exceeding chance levels defined by the shuffled distribution, confirming their ability to learn the reward location in this task (Figure 1B). A previous study performed by blocking dopamine receptors in the hippocampus showed that dopamine was involved in changing reward location information.^12^ Building on this finding, we trained mice to perform a changing reward location task in which only the modification from the fixed reward location task involved randomly changing the position of the reward. Mice maintained an average of 50% correct response rate after 4 days of training, with all wild-type mice exceeding chance levels defined by the shuffled distribution. This indicated that mice could also learn the changing reward location task (Figure 1C). On day 1, the mice could not update the reward location information. However, from day 2 onward, they engaged in exploratory behaviors, nose-poking multiple dispensers to identify the reward location, and gradually selected only one specific reward location. Mice adaptation to reward location rules did not necessarily correlate with the correct response rate. In addition to the correctness, we also calculated the rule-learning rate (i.e., the number of laps in which the animal chose only one of the four available feeders in each lap, regardless of whether it was a reward-providing feeder, divided by the total number of laps) to assess rule-learning abilities. In wild-type mice, the rule learning rate progressively increased over 4 days, ultimately reaching an average of 50%. All mice exceeded the chance levels defined by the shuffled distribution, showing that they could learn the rule of changing the reward location task (Figure 1C).

### Effect of loss of dopaminergic neurons on reward location persistence and updating

**Figure 2:**
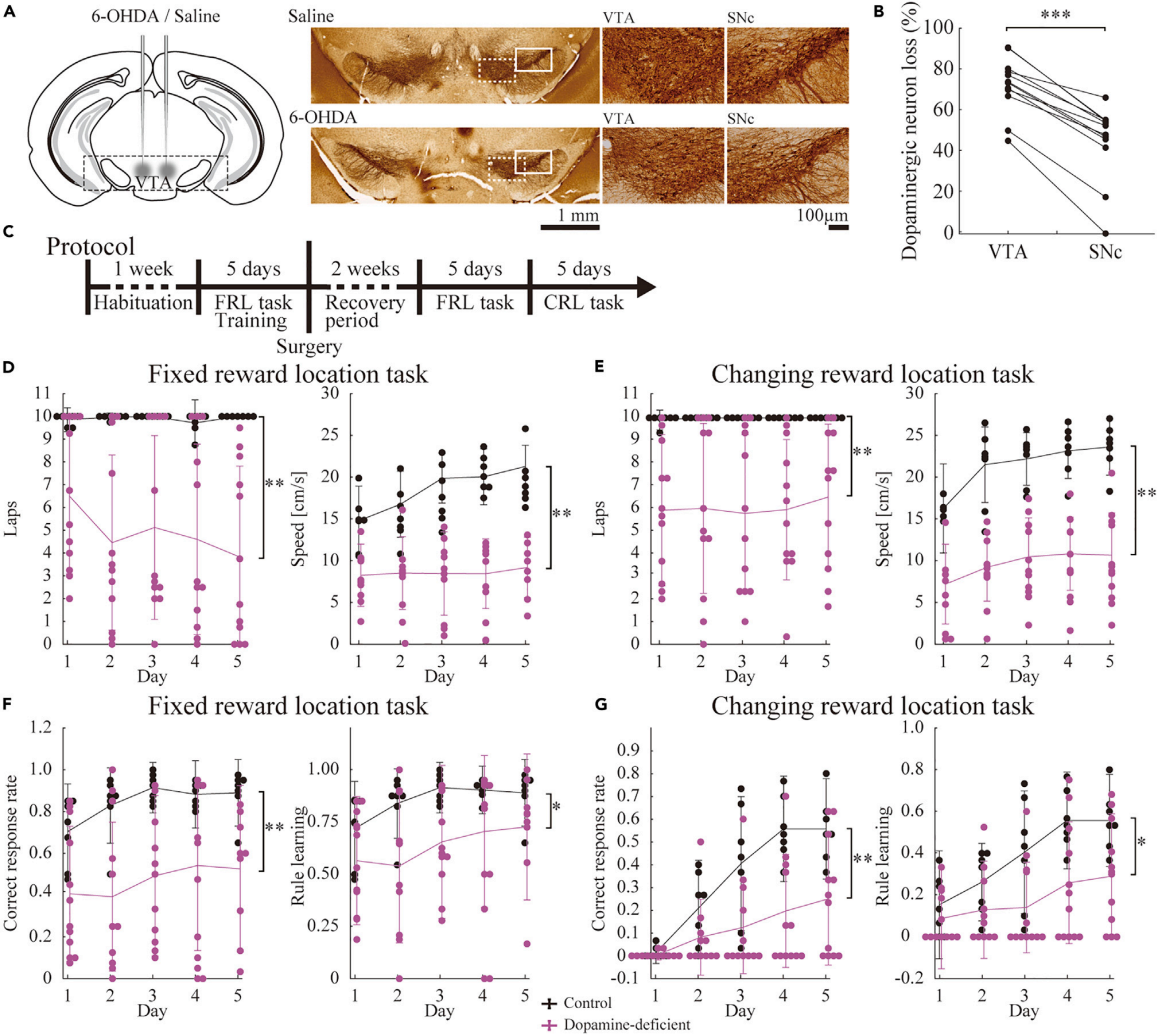
Dopamine deficiency alters task performance and learning in mice (A) Post-hoc verification of bilateral injection of 6-hydroxydopamine (6-OHDA) or saline into the VTA with cannula tips for drug injection (left). Representative tyrosine hydroxylase (TH)-stained coronal sections of saline (top)- or 6-OHDA (bottom)-injected mice are displayed on the right side. The areas within the white solid (SNc) or dotted (VTA) rectangles are presented in magnified views (right). (B) The percentage of dopaminergic neuron loss in the VTA and SNc. ∗∗∗: p < 0.001, Wilcoxon signed-rank test, n = 12 mice. (C) Experimental timeline. Mice are habituated to the maze for 1 week, followed by 5 days of training in the fixed reward location (FRL) task. After a 2-week recovery period post-injection surgery, mice perform the FRL task for 5 days; then the changing reward location (CRL) task for 5 days. (D and E) The number of laps (left) and running speed to reach the reward location (right) during the fixed reward location task (D) or changing reward location task (E) performance for dopamine-deficient (purple) and non-deficient (black) mice. (F and G) Correct response rate (left) and rule learning rate (right) for the fixed reward location task (F) and changing reward location task (G) performance of dopamine-deficient (purple) and non-deficient (black) mice. Dots represent individual data points for each mouse, while the graph indicates mean ± standard deviation (SD). n.s.: p > 0.05, ∗: p < 0.05, ∗∗: p < 0.01, two-way mixed ANOVA. n = 7 mice (control), 12 mice (dopamine deficient).

We selectively lesioned the dopaminergic neurons of mice using the neurotoxin 6-hydroxydopamine (6-OHDA) (Figure 2A) and subjected them to fixed and changing reward location tasks (Figure 2C). Mice administered with saline instead of 6-OHDA were used as controls. Dopaminergic neuron loss in the VTA significantly exceeded that in the substantia nigra pars compacta (SNc) (p < 0.001, Wilcoxon signed-rank test, n = 12 mice; Figure 2B), corroborating the anticipated preponderance of deficiencies in the VTA. However, the non-trivial extent of dopaminergic loss in the SNc warrants consideration in subsequent analyses.

We compared the total number of laps run and running speed to reach the reward location within a session between VTA/SNc dopaminergic neuron-deficient and control mice. Our data highlighted the significant influence of dopaminergic deficiency on these measures in both tasks. In the fixed-reward location task, there were significant variations in both the number of laps (F_1,17_ = 12.8, p < 0.01, Greenhouse–Geisser-corrected) and running speed (F_1,14_ = 26.7, p < 0.01) (Figure 2D). Similarly, in the changing reward location task, marked differences were noted in the number of laps (F_1,17_ = 13.4, p < 0.01, Greenhouse–Geisser-corrected) and running speed (F_1,10_ = 39.4, p < 0.01) (Figure 2E). These results were obtained from a two-way mixed analysis of variance (ANOVA) involving 7 control mice and 12 dopaminergic-deficient mice. The analysis indicated that a deficiency in VTA/SNc dopaminergic neurons significantly altered the number of laps completed and the speed at which the rewards were reached. This finding highlights the importance of dopamine levels from VTA/SNc in maintaining motivation and motor skills necessary for task execution. The fixed reward location task demonstrated significant influences of the experimental day and the interaction between dopamine deficiency and the experimental day on the outcomes of total lap run and running speed (p < 0.05, interaction, Greenhouse–Geisser-corrected). Conversely, the changing reward location task showed no significant interaction effect (p > 0.05, interaction, Greenhouse–Geisser-corrected).

In the fixed reward location task, we found significant discrepancies in both the rates of correct response and rule learning (correct response: F_1,14_ = 9.83, p < 0.01; rule learning: F_1,14_ = 7.57, p < 0.05, Greenhouse–Geisser-corrected; Figure 2F). Moreover, these discrepancies were also evident in the changing-reward location task (correct rate: F_1,16_ = 9.74, p < 0.01; rule learning: F_1,16_ = 7.51, p < 0.05; Figure 2G). These findings, analyzed using a two-way mixed ANOVA, are consistent with the results of previous studies^7^^,^^8^^,^^9^ that showed that a deficiency in VTA/SNc dopaminergic neurons could contribute to the maintenance of spatial memory during the performance of tasks with a fixed reward location. Furthermore, these findings are also consistent with evidence from a previous study, which involves the blockade of hippocampal dopaminergic receptors, that shows that the release of local dopamine in the hippocampus plays a role in updating spatial goals.^12^

### Effect of blockade of hippocampal dopamine receptors

**Figure 3:**
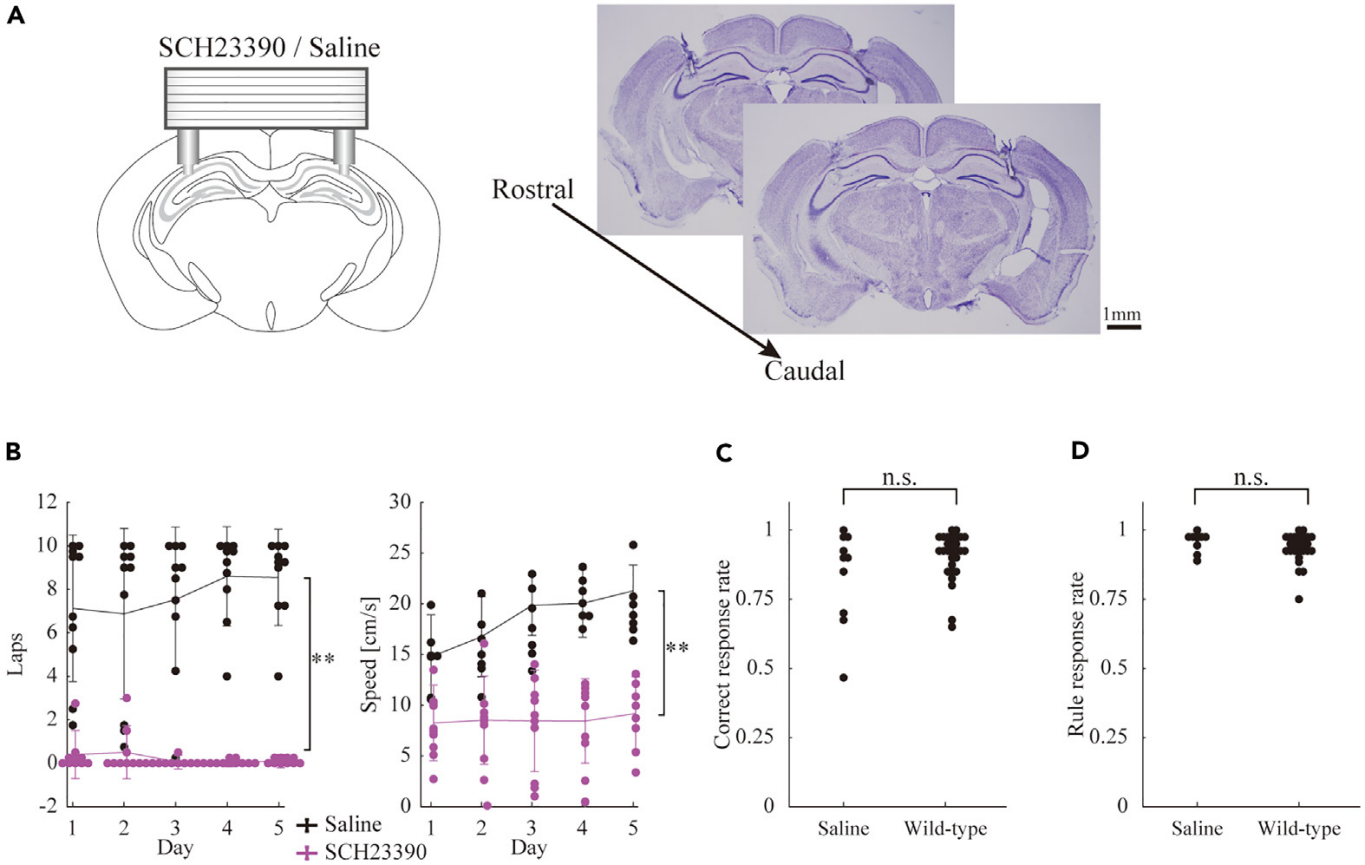
Dopamine receptor blockade induces behavioral abnormalities in mice (A) Schematic representation of the experimental setup for pharmacological experiment. Representative Nissl-stained coronal sections of mice treated with SCH23390 were displayed on the right side. (B) The number of laps during the fixed reward location task performance for dopamine receptor-blockade (purple, SCH23390) and vehicle control (black, saline) mice. Statistical significance is determined using two-way repeated-measures ANOVA. ∗∗: p < 0.01, n = 10 mice. (C and D) Correct response rate (C) and rule learning rate (D) for the fixed reward location task performance of vehicle control (saline) and non-operated wild-type mice. Wilcoxon rank-sum test, n.s.: p > 0.05, vehicle control: n = 10 mice, wild-type: n = 29 mice.

The association between VTA-derived dopamine and the hippocampal response in the context of reward location alterations is challenging to discern because of the significant influences on motivation, motor impairment, or both and the broad projections of VTA/SNc dopaminergic neurons.^15^ This complexity underscores the challenge of making definitive interpretations. To isolate hippocampal-dependent effects from the broader projections of VTA/SNc dopaminergic neurons, we administered SCH23390, a selective dopamine D1 receptor antagonist, to the dorsal hippocampus of mice subjected to a fixed reward location task (Figure 3A). Saline-administered mice served as vehicle controls. Interestingly, mice with D1 receptor blockade remained largely stationary near the feeder. There were significant differences in the total number of laps run within a session between the D1 receptor-blocked mice and the vehicle control mice (F_1,9_ = 89.7, p < 0.01, Greenhouse–Geisser-corrected) (Figure 3B). These findings were derived from a two-way repeated-measures ANOVA involving 10 control and 10 dopamine receptor-blocked mice. Importantly, saline infusion did not impair task performance, as supported by comparable correct response rates and rule-learning metrics between vehicle controls and pre-operated wild-type mice (correct response: p > 0.05; rule learning: p > 0.05, Wilcoxon rank-sum test; Figures 3C and 3D). This unexpected result underscores the intrinsic role of dopamine in the dorsal hippocampus for sustaining the motivation and/or motor skills essential for task performance, independent of the broader VTA/SNc dopaminergic projections. Owing to the near-immobility of the dopamine receptor-blocked mice in the maze, we were unable to assess their performance in tasks involving both fixed and changing reward locations.

### Optogenetic activation of the ventral tegmental area-hippocampal pathway enhances reward location adaptation

**Figure 4:**
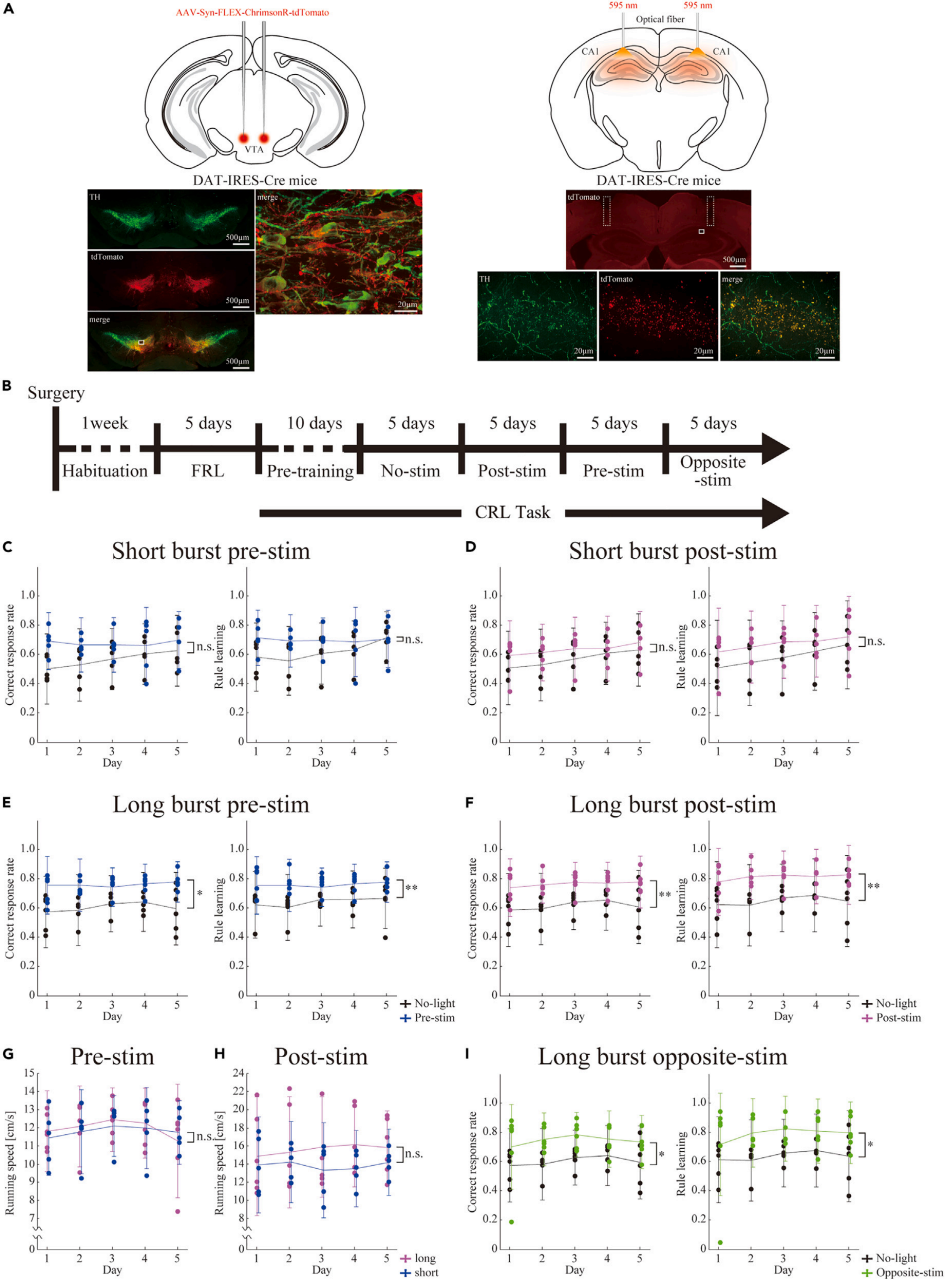
Effects of optogenetic stimulation to the ventral tegmental area (VTA)-hippocampal pathway (A) Schematic representation of the experimental setup for optogenetic stimulation to the dorsal hippocampal CA1 of DAT^VTA^ mice. Injection of AAV-Syn-FLEX-ChrimsonR-tdTomato into the VTA of DAT-IRES-Cre mice generated DAT^VTA^ mice (top, left). Representative injection sites in tyrosine hydroxylase (TH)-stained coronal sections of DAT^VTA^ mice (bottom, left). The co-stained regions (yellow) indicate an overlap between AAV infection (red) and TH-stained areas (green), primarily localized to the VTA. The area within the white rectangle is presented in a magnified view. We proceed with the bilateral insertion of an optical fiber for optogenetic stimulation into the dorsal hippocampal CA1 of DAT^VTA^ mice (top, right). Representative insertion sites in coronal sections of DAT^VTA^ mice (middle, right). The dashed rectangle depicts the tip of the optical fiber. The area within the white rectangle is presented in a magnified view (bottom, right). The co-stained regions (yellow) indicate an overlap between tdTomato expression (red) and TH-stained areas (green). (B) Experimental timeline illustrating the sequence of behavioral tasks and optogenetic stimulation. Mice undergo 1 week of habituation to the maze, followed by 5 days of training in the fixed reward location (FRL) task. Subsequently, mice receive 10 days of pre-training in the changing reward location (CRL) task and then perform the CRL task with various stimulation conditions (no-stim, post-stim, pre-stim, and opposite-stim) for 5 days. (C and D) Correct response rate (left) and rule learning rate (right) for the CRL task with short-duration burst stimulation at the pre-stim (C) or post-stim (D) conditions. (E and F) Correct response rate (left) and rule learning rate (right) for the CRL task with long-duration burst stimulation at the pre-stim (E) or post-stim (F) conditions. (G and H) The running speed to reach the reward location during the CRL task performance at the pre-stim (G) or post-stim (H) conditions. (I) Correct response rate (left) and rule learning rate (right) for the CRL task with long-duration burst stimulation at the opposite-stim condition. Dots represent raw data points for individual mice, while the graph displays the mean ± standard deviation (SD). Statistical significance is determined using two-way mixed ANOVA. n.s.: p > 0.05, ∗: p < 0.05, ∗∗: p < 0.01.

Dopamine inputs to the hippocampus originate from both the VTA/SNc and LC.^9^^,^^16^ Thus, considering our findings, focusing on the specific activation, rather than inhibition, of the input from VTA dopaminergic neurons to the hippocampus is crucial.^8^ We examined the causal relationship between VTA dopaminergic neurons expressing the dopamine transporter (DAT) and the dorsal hippocampus. A Cre-inducible viral construct encoding ChrimsonR^17^ fused to an enhanced red fluorescent protein (ChrimsonR-tdTomato) was injected into the VTA of DAT-IRES-Cre mice (Figure 4A). A fiber-optic tip was positioned directly above the pyramidal cell layer of the dorsal hippocampal CA1 of DAT^VTA^ mice, and orange light was applied as an intervention. The data without light exposure were used as controls for the same group of mice.

We applied two types of burst photostimulation for 1 s with a long (40 ms) or short (10 ms) duration and 20-Hz pulse pattern during the changing reward location task at three different times: pre-stim, that is, after the infrared-blocking sensor situated 15 cm in front of the reward location was passed; post-stim, that is, 1 s after nose-poking at the reward location; and opposite-stim, that is, after the infrared-blocking sensor situated 15 cm in front of the opposite side of the reward location (e.g., south when the reward location was north) was passed (Figure 4B).

Short burst stimulation at pre-stim and post-stim did not significantly affect either the correct response rate or rule learning rate (Figures 4C and 4D; pre-stim: correct response: F1,4 = 7.10, p > 0.05, rule learning: F1,4 = 7.17, p > 0.05; post-stim: correct response: F1,4 = 5.51, p > 0.05, rule learning: F1,4 = 5.51, p > 0.05, two-way repeated-measures ANOVA, n = 5 mice; Videos S1 and S2). In contrast, long burst stimulation at pre-stim and post-stim led to the learning of the fast adaptation to the changed reward location from the first day, and there were significant differences in both the correct response and rule learning rates compared with those of the control group (Figures 4E and 4F; pre-stim: correct response: F1,5 = 9.97, p < 0.05, interaction: p > 0.05, rule learning: F1,5 = 17.4, p < 0.01, interaction: p > 0.05, Greenhouse–Geisser-corrected; post-stim: correct response: F1,5 = 19.0, p < 0.01, interaction: p > 0.05, rule learning: F1,5 = 22.4, p < 0.01, interaction: p > 0.05, Greenhouse–Geisser corrected, two-way repeated-measures ANOVA, n = 6 mice; Videos S3 and S4). This timing-invariant effect implies that this involvement is independent of internal events around the reward location, such as hippocampal reward cells or place cell activity. Furthermore, there was no significant difference in running speed toward the reward location between the short- and long-duration burst stimulations, regardless of whether the conditions were pre-stim or post-stim (Figures 4G and 4H; pre-stim: F1,7 = 0.0251, p > 0.05; post-stim: F1,9 = 0.781, p > 0.05; two-way mixed ANOVA). These findings show that the rapid goal adaptation effect is not solely attributed to heightened motivation or motor activity around the reward location caused by the long-duration burst stimulation of the VTA-hippocampal dopaminergic pathway.

Video S1. Representative task performance with short pre-stim (reward: north), related to Figure 3

Video S2. Representative task performance with long pre-stim (reward: west), related to Figure 3

Video S3. Representative task performance with short post-stim (reward: east), related to Figure 3

Video S4. Representative task performance with long post-stim (reward: west), related to Figure 3

One well-established role of VTA dopaminergic neurons is to encode reward prediction errors. Interestingly, the long-duration burst stimulation of opposite-stim also led to significant outperformance in the changing reward location task (opposite-stim: correct response: F1,5 = 8.76, p < 0.05, interaction: p > 0.05; rule learning: F1,5 = 7.88, p < 0.05, interaction: p > 0.05, Greenhouse–Geisser-corrected, two-way repeated-measures ANOVA, n = 6 mice; Figure 4I and Video S5). This location-invariant effect shows that the enhancement is independent of the reward prediction signals but solely dependent on the activation of VTA dopaminergic inputs.

Video S5. Representative task performance with long opposite-stim (reward: north), related to Figure 3

### Distinct hippocampal responses to burst stimulation in the ventral tegmental area-hippocampal pathway

**Figure 5:**
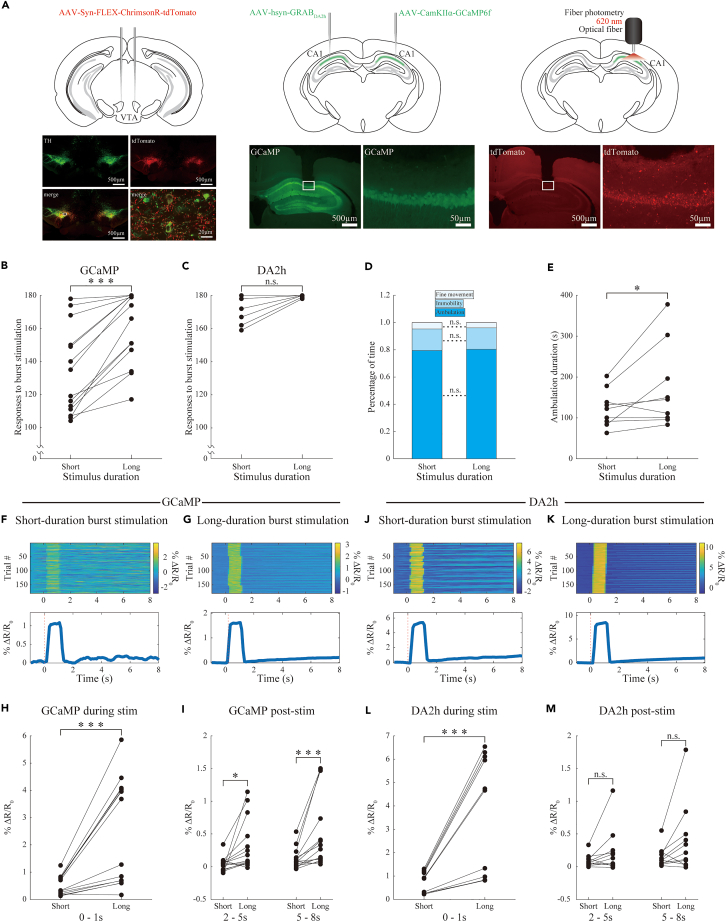
Hippocampal response diversity to burst stimulation of ventral tegmental area (VTA) dopaminergic axons (A) Schematic representation of the experimental setup for concurrent optogenetic and fiber photometry investigation. Bilateral injection of AAV-Syn-FLEX-ChrimsonR-tdTomato into the VTA of DAT-IRES-Cre mice generated DAT^VTA^ mice (top, left). Representative injection sites in tyrosine hydroxylase (TH)-stained coronal sections of DAT^VTA^ mice (bottom, left). The co-stained regions (yellow) indicate an overlap between tdTomato expression (red) and TH-stained areas (green), primarily localized to the VTA. The area within the white rectangle is presented in a magnified view. Unilateral injection of AAV-CamKIIα-GCaMP6f and/or AAV-hsyn-GRAB_DA2h_ into the dorsal hippocampus of the DAT^VTA^ mice was performed (top, middle). Representative GCaMP6f expression in the coronal section of the dorsal hippocampus of the DAT^VTA^ mice (bottom, middle). Calcium or dopamine signals are recorded via unilateral insertion of an optical fiber into the dorsal hippocampal CA1 of the DAT^VTA^ mice while simultaneously stimulating the VTA dopaminergic axons (top, right). Representative tdTomato expression in the coronal section of the dorsal hippocampus of DAT^VTA^ mice (bottom, right). (B and C) The count of GCaMP6f (B) or GRAB_DA2h_ (C) responses to burst stimulations that exceed the predetermined threshold level as a function of the duration of stimulation. Wilcoxon signed-rank test, ∗∗∗: p < 0.001, n.s.: p > 0.05, GCaMP6f: n = 14 mice, GRAB_DA2h_: n = 11 mice. (D) Percentage of time of motor behavioral events (ambulation, immobility, and fine movement) as a function of the duration of stimulation (short or long). Multiple Wilcoxon signed-rank test with Bonferroni correction, n.s.: p > 0.05. n = 9 mice. (E) The graphs illustrate the ambulation duration as a function of the duration of stimulation. Wilcoxon signed-rank test, ∗: p < 0.05, n = 9 mice. (F and G) Representative % ΔR/R_0_ calcium signals from a mouse during individual trials (top) and averaged across trials (bottom) during short (F) and long (G) burst stimulations. Individual signal intensity is color coded, as detailed in the color bar on the right. (H and I) Average % ΔR/R_0_ calcium signals during stimulation (0–1s) (H) or early (2–5s) or late (5–8s) phase of post-stimulation (I) as a function of the duration of stimulation (short or long) (n = 14 mice). (J and K) Representative % ΔR/R_0_ dopaminergic responses from a mouse during individual trials (top) and averaged across trials (bottom) during short (J) and long (K) burst stimulations. Individual signal intensity is color coded, as detailed in the color bar on the right. (L and M) Average % ΔR/R_0_ dopaminergic responses during stimulation (0–1s) (L) or early (2–5s) or late (5–8s) phase of post-stimulation (M) as a function of the duration of stimulation (short or long) (n = 11 mice). Wilcoxon signed-rank test, ∗: p < 0.05, ∗∗∗: p < 0.001, n.s.: p > 0.05.

Finally, to isolate the impact of stimulation duration on hippocampal activity, we examined how axonal excitation of VTA dopaminergic neurons influenced the activity of hippocampal CA1 pyramidal cells of freely roaming DAT^VTA^ mice in an open field using fiber photometry, thereby eliminating task-related confounds. An adeno-associated virus (AAV) vector containing a CaMKIIα promoter construct encoding GCaMP6f was injected into the pyramidal cells of the dorsal hippocampal CA1 (Figure 5A). The excitation level during the long-duration burst pattern was consistently higher throughout the entire 180-s stimulation period than the 1-s pre-stimulation level. In contrast, the short-duration burst pattern, commonly employed in previous studies,^8^^,^^16^ produced a less stable effect (Figures S1A and S1B). There was a significantly higher count of responses exceeding the predetermined threshold level for long-duration burst stimulations than for short-duration stimulations (p < 0.001, Wilcoxon signed-rank test, n = 14 mice; Figure 5B). To analyze the corresponding behavioral outputs, the behavior was categorized into three groups based on their velocities: ambulation, immobility, and fine movement. There were no significant differences in the distribution of these behavioral periods (p > 0.05, multiple Wilcoxon signed-rank test with Bonferroni correction, n = 9 mice; Figure 5D). Most events involved ambulation; thus, we further analyzed the duration of ambulation and the findings showed that the long-duration burst pattern extended ambulation duration (p < 0.05, Wilcoxon signed-rank test, n = 9 mice; Figure 5E). For calcium signaling in response to short- and long-duration burst stimulations (Figures 5F and 5G), there were significant differences across all examined time windows (during stimulation: p < 0.001; 2–5s post-stimulation: p < 0.01; 5–8s post-stimulation: p < 0.001; Wilcoxon signed-rank test, n = 14 mice, Figures 5H and 5I).

To specifically gauge dopaminergic activity, we used an AAV vector encoding the GRAB_DA2h_ sensor^18^ under a human synapsin promoter and targeted the dorsal hippocampal CA1. Unlike the pyramidal cell activity, the excitation level of dopamine responses during the short- and long-duration burst pattern was consistently higher throughout the entire 180-s stimulation period than those in the 1-s pre-stimulation period (Figures S1C and S1D). Responses exceeding the predetermined threshold for long-duration burst stimulations were not significantly higher than that for short-duration stimulations (p > 0.05, Wilcoxon signed-rank test, n = 11 mice; Figure 5C). For dopaminergic signals elicited by short- and long-duration bursts (Figures 5J and 5K), there were significant differences during stimulation (p < 0.001, Wilcoxon signed-rank test) but not after stimulation (2–5s and 5–8s post-stimulation: p > 0.05; Wilcoxon signed-rank test) (n = 11 mice, Figure 5L–5M).

## Discussion

The hippocampus plays a crucial role in cognitive map formation. This is supported by the identification of place cells that are integral to determining goal locations,^19^^,^^20^^,^^21^ particularly in the context of spatial navigation. The mesolimbic dopaminergic system is an essential modulator of various learning processes. Recent evidence regarding hippocampal reward cells indicate that dopaminergic input from the VTA to the hippocampus contributes to the persistence and adaptation of reward-related cognitive maps.^8^^,^^11^ However, a comprehensive understanding of the causal relationship between hippocampal dopaminergic inputs and the rapid adaptation to this goal remains to be elucidated.

Our study establishes that VTA dopaminergic neuron lesions significantly impair the ability to reliably recall reward locations, consistent with prior evidence of VTA and LC dopaminergic axonal effects in the hippocampus.^6^^,^^8^^,^^9^ Notably, the accompanying motor or motivational deficits persisted even when dopamine receptors in the dorsal hippocampus were selectively inhibited. This suggests that mesolimbic VTA dopaminergic pathways, independent of the hippocampus, are not the sole regulators of motor or motivational impairments. Furthermore, our findings support that hippocampal dopamine-induced deficits in task performance may further contribute to spatial memory impairments. Thus, the targeted modulation of VTA dopaminergic input to the hippocampus is a pivotal factor in understanding the association between hippocampal dopaminergic signaling and the dynamic adaptation of goal locations.

Our observations regarding the swift adaptation of goal locations can be analyzed through the prism of four distinct hypotheses. The timing hypothesis underscores that the timing of neural excitation, especially in conjunction with reward receipt, is a key determinant of cognitive performance optimization.^22^ The training hypothesis postulates that recurrent patterns of neural excitation serve as a form of neural “conditioning,” aiding in overcoming hurdles that impede the translation of learned knowledge into behavior.^23^^,^^24^ The threshold hypothesis proposes that a certain degree or threshold of neural excitation is a prerequisite for cognitive enhancement.^25^ Finally, the specificity hypothesis asserts that particular subsets of neurons or their connections within the VTA–hippocampal dopaminergic pathway are vital for cognitive enhancement.^2^^,^^15^

We observed swifter adaptation to changes in reward location when using optogenetic stimulation that precisely targeted VTA dopaminergic inputs to the dorsal hippocampus. This swift adaptation was evident in the consistency and specificity of goal adaptation, unlike in non-stimulated inputs. Our findings challenge the idea that artificial stimulation in proximity to the food dispenser could inadvertently trigger the cessation of movement and subsequent swift shift in the reward location. Notably, a significant enhancement in goal adaptation was observed even when an identical stimulation was applied at a position not typically associated with the reward location, known as the opposite-stim condition. Furthermore, long-duration burst stimulation of the VTA-hippocampal dopaminergic pathway appeared to facilitate ambulation. These observations show that activation operates independently of reward-related processes, such as reward prediction error signals. Therefore, the significance of the timing of neural excitation, as shown by the timing hypothesis, may be less critical than previously assumed. However, it may still contribute to the overall cognitive performance in a more nuanced manner.

Interestingly, the rapid response to changes in goal locations indicated a marked improvement in performance compared to non-stimulated conditions. This observation shows that the VTA dopaminergic input into the hippocampus may act as a “teaching signal” to enhance cognitive performance, as proposed by the training hypothesis. However, this hypothesis does not fully explain the observed improvement in the opposite-stim condition. This finding shows that while mice appear capable of quickly learning changes in the updated goal location, they may encounter resistance when attempting to translate this acquired knowledge into behavior. It is plausible that the VTA dopaminergic inputs alleviate this resistance, thereby facilitating the expression of learned behaviors. Future research should explore the potential factors contributing to this resistance, including synaptic plasticity, neuromodulation, and various intracellular signaling pathways.^10^

Our current study presents a contrasting perspective to that of previous findings that explored the role of VTA dopaminergic inputs within the dorsal hippocampus in spatial memory persistence and reactivation.^8^ Our results revealed that the short-duration burst optogenetic stimulations used in the previous study were insufficient to drive rapid goal adaptation. However, it is crucial to highlight not only the stark contrast in light intensities, but also the distinct optogenetic tools employed: 10–20 mW via laser for ChR2^8^ versus only 2.5 mW via LED for ChrimsonR in our investigation. This supports the idea that the VTA–hippocampal dopaminergic pathway exhibits multifaceted functions modulated by distinct activation patterns and/or intensity, as indicated by the threshold hypothesis.

The observed improvement in spatial goal adaptation cannot be solely attributed to dopamine release. It may also involve the co-release of neurotransmitters such as glutamate or gamma-aminobutyric acid (GABA) from VTA dopaminergic axon terminals.^26^^,^^27^ This assertion is supported by the significant increase in excitability of hippocampal pyramidal cells during the long-duration burst stimulation of VTA-derived axons—a response that persists post-stimulation, unlike the more transient dopamine signals. This suggests that not only dopamine, but also co-released glutamate and/or GABA contribute to the enhanced spatial goal adaptation. Although this study does not delve into this concept, future research could compare outcomes by stimulating axons in other projection targets of VTA dopaminergic neurons. Moreover, applying the cell type-specific inhibition of VTA dopaminergic neuron activity could elucidate the contributions of each projection site, thereby providing a comprehensive understanding of the underlying neural mechanisms and supporting the specificity hypothesis.

A previous study used dopamine receptor blockers and electrophysiological methods to show a relationship between the formation of goal-related cognitive maps in the hippocampus and local dopamine release. This study found that when the reward location was changed without dopamine, the remapping of place fields was inhibited.^12^ According to this notion, exciting the axons of the VTA dopaminergic neurons in the hippocampus may facilitate the induction of place-field remapping. In contrast, as seen in previous studies on reward cells, place cells may not undergo place field remapping; instead, only the place field of the reward cells might rapidly shift.^11^ Our study methodology cannot address these questions. However, our results emphasize the importance of VTA dopaminergic neuron excitation in the hippocampus for adaptation to changes in reward locations during goal-directed navigation.

In summary, our study provides valuable insights into the role of dopaminergic inputs in the dorsal hippocampus in facilitating the rapid adaptation of goal locations during spatial navigation. However, further research is needed to develop a more comprehensive understanding of the neural mechanisms involved. This includes exploring the contributions of other neurotransmitter systems, the potential role of place field remapping, and the specific functions of distinct activation patterns within the VTA-hippocampal dopaminergic pathway. We can advance our knowledge of the neural substrates underlying goal-directed navigation and cognitive map formation by addressing these limitations and building on the current findings.

### Limitations of the study

In this study, optogenetic activation of VTA axons in the hippocampus facilitated rapid adaptation to changing goal locations. However, provided that the antidromic spikes generated by photostimulation propagate back to VTA dopaminergic neurons, thereby affecting other brain regions to which these neurons project, the potential for antidromic action potentials complicates the interpretation of these results. Additionally, the dopaminergic input to the hippocampus is not exclusively VTA derived; it also originates from the LC, adding another layer of complexity to our findings. Our results, particularly concerning dopamine receptor antagonist experiments, diverge from prior studies. We observed behavioral abnormalities upon administering a dopamine receptor antagonist in the hippocampus, a finding not reported in previous studies employing similar pharmacological methodologies.^12^^,^^28^ This discrepancy may be due to differing experimental paradigms: an open field in prior studies versus an elevated maze in ours, which introduces anxiety-like behavior.^29^^,^^30^ Such emotional contexts could significantly influence outcomes and may explain the difference from previous findings. Given these complexities and controversies, new experimental designs are essential for a nuanced understanding of the mechanisms involved. Future research should consider emotional context, compare the role of dopaminergic inputs between LC and VTA, and investigate the potential impact of antidromic spikes on other projection targets of VTA dopaminergic neurons.

## STAR★Methods

### Key resources table

**Table:** 

REAGENT or RESOURCE	SOURCE	IDENTIFIER
**Antibodies**		

mouse anti-tyrosine hydroxylase	Merck Millipore	Cat#MAB318; RRID:AB_2313764
rabbit anti-tyrosine hydroxylase	Merck Millipore	Cat#AB152; RRID:AB_390204
biotinylated donkey anti-mouse IgG	Jackson ImmunoResearch Inc.	Cat#715-065-151; RRID:AB_2340785
Alexa Fluor 488-conjugated cross-adsorbed goat anti-rabbit IgG (H + L)	Thermo Fisher Scientific	Cat#A11008; RRID:AB_143165

**Bacterial and virus strains**		

pAAV-Syn-FLEX-rc [ChrimsonR-tdTomato]	Klapoetke et al., 2014^17^	Cat#62723-AAV5; RRID:Addgene_62723
pENN.AAV.CamKII.GCaMP6f.WPRE.SV40	Penn Vector Core Addgene	Cat#100834-AAV1; RRID:Addgene_100834
pAAV-hsyn-GRAB_DA2h_	Sun et al., 2020^18^	Cat#140554-AAV9; Addgene_140554

**Chemicals, peptides, and recombinant proteins**		

6-hydroxydopamine	Sigma-Aldrich	Cat#162957
SCH23390	Sigma-Aldrich	Cat#D054

**Deposited data**		

data, custom-written software, and 3D models	This paper	https://doi.org/10.5281/zenodo.8409826

**Experimental models: Organisms/strains**		

Mouse: C57BL/6J	Jackson Laboratory	stock number: 000664
Mouse: DAT Cre recombinase (DAT-IRES-Cre)	Jackson Laboratory	stock number: 006660

**Software and algorithms**		

DeepLabCut	Mathis et al., 2018^31^	GitHub - DeepLabCut/DeepLabCut: Official implementation of DeepLabCut: Markerless pose estimation of user-defined features with deep learning for all animals incl. humans
pMAT	Bruno et al., 2020^32^	GitHub - djamesbarker/pMAT: Fiber Photometry Modular Analysis Tool Suite

### Resource availability

#### Lead contact

Further information and requests for resources and reagents should be directed to and will be fulfilled by the lead contact, Susumu Takahashi (stakahas@mail.doshisha.ac.jp).

#### Materials availability

No newly generated materials are associated with this paper.

#### Data and code availability

All data, custom-written software, and 3D models used in this study are available from the Zenodo repository (https://doi.org/10.5281/zenodo.8409826).

### Experimental model and study participant details

#### Animals and housing conditions

Male and female DAT Cre recombinase (DAT-IRES-Cre) mice (Jackson Laboratory, stock number 006660)^33^ and C57BL/6J mice aged approximately 6 months at the start of the experiments were used. The mice were individually housed in separate cages, with environmental conditions maintained at a temperature range of 24°C–26°C and a consistent 12-h light/dark cycle. Experimental procedures were performed exclusively during the light phase. The body weight of each animal was adjusted to approximately 80% of their respective body weight *ad libitum*. While unrestricted access to water was provided, food intake was carefully controlled. Cages and housing conditions were refreshed weekly. All procedures in this study were approved by the Institutional Animal Care and Use Committee of Doshisha University.

### Method details

#### Surgical procedures

For the lesion experiment, the mice were anesthetized using isoflurane (2%, Mylan EPD Inc., PA, USA), and a 6-OHDA solution (4 mg/mL; Sigma-Aldrich, 162957) was prepared in 0.1% L-ascorbic acid dissolved in saline (Otsuka Normal Saline, Otsuka Pharmaceutical Factory, Japan). The 6-OHDA solution was bilaterally injected at a volume of 0.5 μL into the VTA through the pre-implanted cannula at stereotaxic coordinates: AP: −3.1 mm, ML: ±0.4 mm, and DV: −4.2 mm. Following injection, the animals were allowed a recovery period of at least 1 week before conducting the post-lesion behavioral tests. In the control group, saline was bilaterally injected into the VTA using the same procedure as that used for 6-OHDA injection. For the micro-infusion experiment, mice were anesthetized with isoflurane to selectively block the dopamine D1 receptor in the dorsal hippocampus. To implant a cannula in both hemispheres of the dorsal hippocampus, two cannulas (diameter 22G, 5.0 mm long, PlasticsOne, TX, USA) were bilaterally placed at coordinates AP: −2.4 mm, ML: ±2.5 mm, and DV: −1.4 mm using a bilateral internal cannula (C232G, PlasticsOne, TX, USA).

For the optogenetics experiment, to selectively activate the axons of the VTA dopaminergic neurons projecting to the dorsal hippocampus, mice were anesthetized with isoflurane, and six holes were drilled into their skulls for anchor-screw placement. The anchor screws were then installed. Additionally, two holes were made in the VTA for AAV administration, followed by bilateral injection of 0.8 μL AAV (pAAV-Syn-FLEX-rc [ChrimsonR-tdTomato], titer 8.5 × 10^12^ genome copies/mL, Addgene, 62723-AAV5, MA, USA) at each site. To implant an optical fiber in both hemispheres of the dorsal hippocampus, two optical fibers (FT200EMT, diameter 200 μm, pure silica material, THORLABS, NJ, USA) were bilaterally placed at coordinates AP: −2.0 mm, ML: ±1.9 mm, and DV: −0.8 mm using a custom adapter designed in-house using Onshape (Parametric Technology Corporation, MA, USA) and printed with a 3D printer (Form 2/3, Formlabs Inc., MA, USA).

For concurrent optogenetic and fiber photometry experiments, 0.8 μL AAV (pAAV-Syn-FLEX-rc [ChrimsonR-tdTomato], titer 8.5 × 10^12^ genome copies/mL, Addgene) was bilaterally injected into both hemispheres of the VTA. In addition, 0.8 μL AAV (pENN.AAV.CamKII.GCaMP6f.WPRE.SV40, titer 8.5 × 10^12^ genome copies/mL, or pAAV-hsyn-GRAB_DA2h_, titer 8.5 × 10^12^ genome copies/mL, Addgene, 100834-AAV1 or 140554-AAV9) was injected into the unilateral (GCaMP6f or GRAB_DA2h_) or bilateral (GCaMP6f on one side and GRAB_DA2h_ on the other) hippocampal CA1 (AP: −2.0 mm, ML: ±2.0 mm, and DV: −1.1 mm) for fiber photometry. This was followed by implantation of a TeleFiOpto cannula (diameter 400 μm, Bio Research Center, Co., Ltd.) and fixation with dental glue (RelyX Unicem 2, 3M, MN, USA). The surface of the glue was black-coated with dental cement kneaded with carbon powder (Sigma-Aldrich, 484164) to prevent the optogenetically stimulated light and fiber photometry excitation light from leaking through the translucent glue.

#### Apparatus

A reconfigurable maze was used for behavioral testing, as described in a previous study.^34^^,^^35^ The maze contained various objects such as mushroom-shaped, cylindrical, and triangular prisms. A sensor system was integrated to record the passage and poking timing of the mice, which were then sent to a computer via serial communication.

#### Behavioral testing environment

A total of 54 mice were assigned to groups: control, dopamine-deficient, pharmacological intervention, optogenetic stimulation, and concurrent optogenetics and fiber photometry. The control group consisted of 4 C57BL/6J mice and 3 DAT-IRES-Cre mice; the dopamine-deficient group, 12 C57BL/6J mice; the pharmacological intervention group, 10 C57BL/6J mice; the optogenetic stimulation group, 8 DAT-IRES-Cre mice (2 and 3 mice used for short and long stimulation, respectively, and 3 mice used for both short and long stimulations); and the concurrent optogenetic and fiber photometry group, 17 DAT-IRES-Cre mice (6 mice expressing GCaMP6f unilaterally, 3 mice expressing GRAB_DA2h_ unilaterally, and 8 mice with GCaMP6f expressed on one side and GRAB_DA2h_ on the other). For the optogenetic stimulation group, unfortunately, due to an incident where mice fell from the elevated maze, the optical fiber adapter sustained damage, rendering the continuation of the experiment impossible.

All behavioral experiments were conducted in a soundproof room with minimal human involvement during the experiments. A 10 mg AIN-76A Rodent Tablet (Test Diet, O’HARA & CO., LTD., Japan) was used as a food reward. Feces and urine were removed between sessions, and maze pathways were lightly cleaned with 70% ethanol. A camera was installed above the maze to record the behavior of the mice during the task.

#### Behavioral paradigms

Behavioral experiments were performed using a reconfigurable maze platform. The maze comprised 8 paths, 4 linear and 4 curved, each measuring 4 cm in width and 40 cm in length, forming a circular maze with a total length of 320 cm. The feeding stations were positioned in the north, south, east, and west. Infrared-blocking sensors were installed 15 cm from each feeding station. The passage through which the mouse traveled was identified based on the sensor data. Transparent acrylic gates were installed between the passages near the feeding stations to prevent the mice from retracing their steps after obtaining food. Three distinct landmark objects were placed within the circular maze: a mushroom-shaped object (an upper hemisphere with a diameter of 18 cm and a lower cylinder with a diameter of 8 cm and a height of 13 cm), a rectangular prism (11 cm × 11 cm × 28 cm), and a triangular prism (an equilateral triangle with 18 cm sides and a height of 24 cm).

The opening and closing of the gates and food administration were controlled using a microcontroller (Arduino MEGA 2560). The Arduino temporarily stored the timings of sensor blockage and nose poking at the feeding stations, simultaneously transferring the data to a host computer via serial communication. The mice were trained in two types of behavioral tasks, namely, a fixed-reward location task and a changing-reward location task, which involved altering the placement of rewards or landmark objects. In both tasks, the start position remained consistent throughout the experiment, and the mice were trained to run in a clockwise direction. Before undertaking the fixed or changing reward location tasks, the mice were first habituated to the maze for 1 week, followed by 5 days of training for the fixed reward location task. One session was defined as either 10 laps of the circular maze or 15 min from the start, whichever came first. The session was terminated when the mouse escaped the maze. In the optogenetic experiments, one session was defined as the completion of 10 laps of the circular maze by the mouse.

#### Fixed reward location task

Throughout the experiment, the reward location remained constant across sessions, whereas the arrangement of landmark objects placed within the maze was randomly altered.

#### Changing reward location task

In each session, the reward location shifted with changes in the arrangement of landmark objects. Adjustments were made to ensure no associations existed to avoid any preserved relative relationship between landmark objects and reward locations when randomly changing positions.

#### Infusion protocol

SCH23390 (D054, Sigma-Aldrich, 5 mg/mL concentration diluted in saline) or saline was infused using a Hamilton syringe (88411, Hamilton, CA, USA) coupled with a motorized stereotaxic microinjector (IMS-20, Narishige, Japan). The infusion was administered bilaterally at a rate of 0.05 μL/min, with a total volume of 0.6 μL per hemisphere (1.2 μL bilaterally) according to previous studies.^12^^,^^28^ The cannula was removed 1 min post-infusion, and the task was initiated 20 min after the cessation of anesthesia.

#### Optogenetic stimulation protocol

For optogenetic stimulation during a task involving dynamic reward locations, light with a wavelength of 595 nm was emitted at a frequency of 20 Hz at ∼2.5 mW/mm^2^ at the fiber implant tip, employing a duty cycle of 4/5 for 1s from the tip of the optical fiber situated directly above the CA1 cell layer within the dorsal hippocampus (light source: Fiber-Coupled LED 595 nm [M595F2]; THORLABS). The experiments were conducted using no light (no-stim) and three distinct light exposure timings (pre-stim, post-stim, and opposite-stim).

##### Pre-stim

Illumination commenced immediately after the mouse traversed the infrared-blocking sensor positioned 15 cm in front of the reward location.

##### Post-stim

Illumination ensued directly after the mouse performed a nose poke to obtain the reward.

##### Opposite-stim

Illumination was initiated immediately after the mouse traversed the infrared-blocking sensor positioned 15 cm before the feeder opposite to the reward location (e.g., the south feeder when the north feeder served as the reward location).

#### Concurrent optogenetics and fiber photometry protocol

Fiber photometry was performed using a wireless fiber photometry and optogenetics system (*TeleFiOpto*; Bio Research Center Co., Ltd., Japan). For optogenetic stimulation, light with a wavelength of ∼620 nm was delivered at a frequency of 20 Hz with 10 ms or 40 ms pulses at ∼1 mW/mm^2^ at the fiber implant tip in 10s sessions consisting of 1s of stimulation followed by 9s of no stimulation. Simultaneously, the mice freely roamed in an open field (diameter: 31 cm, height: 17 cm). This protocol was repeated for 30 min, comprising 180 sessions. Fiber photometry recordings were used to acquire fluorescence signals at an excitation wavelength of approximately 470 nm during optogenetic stimulation.

#### Analysis

Timestamps about the passage of infrared-blocking sensors and nose poke timings measured during the behavioral tasks were temporarily stored on an Arduino microcontroller. After completing all tasks, the data were transferred to a computer via serial communication. Communication between the Arduino microcontroller and the PC was facilitated using MATLAB (Mathworks, MA, USA). For the lesion experiments, the correct response rate, rule learning rate, and number of laps were calculated for each session. The correct response rate was defined as the number of laps in which the animal only chose a reward-providing feeder divided by the total number of laps. The rule learning rate was defined as the number of laps in which the animal chose only one of the four available feeders in each lap, regardless of whether it was a reward-providing feeder, divided by the total number of laps. Four sessions were conducted daily, and the average values were calculated for each subject. In the optogenetic experiments, the correct response and rule learning rates were calculated in the same manner as that in the lesion experiments. In the post-stim trials, the correct response and rule learning rates were calculated starting from the lap following reward acquisition. The running speed was determined by dividing the fixed distance (15 cm) by the time elapsed for each mouse to cross the sensor and poke its nose at the reward location.

Fiber photometry data were recorded using *TeleFipho* software (BioResearch Center, Co., Ltd., Japan) at a sampling rate of 100 Hz to capture both raw photometry data and event data for optogenetic stimuli. These data were analyzed using *pMAT*^32^ to obtain the % ΔR/R_0_ and Z-scores of the peri-event time histograms across the 180 experimental sessions. The time window for this analysis was 1s before and 8s after the start of the stimulation, with a baseline sampling window of 1s immediately before the start of the stimulation and a bin constant of 0.1s. The average % ΔR/R_0_ of GCaMP6f and GRAB_DA2h_ responses by stimulus duration was calculated during stimulation (1s from the onset of the stimulation) and early (2–5s after the onset of the stimulation) and late (5–8s after the onset of the stimulation) phases of post-stimulation, respectively. We also counted the number of experimental sessions where the *Z* score exceeded 1.96 during the 1.5s epoch commencing 0.05s after the start of the stimulation.

Behavioral event classification was performed using concurrent optogenetic and fiber photometry experiments. Using *DeepLabCut*™,^31^ we precisely tracked six joint locations in mice, notably in the neck. Our study primarily utilized neck-point data to distinguish between behavioral events. We defined ambulation events as those when the neck point-derived speed exceeded an average of 2 cm/s for a sustained half-second.^36^ Conversely, immobility events emerged during continuous periods with speeds below 2 cm/s lasting for at least 1s. The remaining movements that did not fulfill the ambulation or immobility criteria were classified as “fine movement events,” reflecting their nuanced nature.

#### Histological assessment

After mice were euthanized through a pentobarbital sodium overdose and subsequently perfused with formalin, their brains were frozen and sectioned coronally at 40-μm thickness using a sliding microtome. The sections were divided into six interleaved sets for immunostaining or Nissl staining with cresyl violet (Sigma-Aldrich, St. Louis, MO, USA; C5042-10G). Free-floating sections were pretreated with 3% hydrogen peroxide and incubated overnight with a primary mouse anti-tyrosine hydroxylase antibody (1:1000; Merck Millipore, MAB318) to assess the dopaminergic neuronal loss. The sections were incubated with the secondary antibody biotinylated donkey anti-mouse IgG (1:100; Jackson ImmunoResearch Inc., 715-065-151), followed by incubation with an avidin-biotin-peroxidase complex solution (1:100; Vector Laboratories, PK6100). Immunoreactivity was visualized using 3-3′ diaminobenzidine tetrahydrochloride (Dojindo Laboratories, 349–00903). The degree of dopaminergic cell loss was estimated by comparing cell counts across representative sections of the VTA and SNc – the most rostral, caudal, and intermediate sections – between lesioned and non-lesioned control mice. The cells were manually counted using a microscope, and images were acquired for further analysis. The acquired images were processed and analyzed using ImageJ or Adobe Photoshop software for cell identification and quantification. Free-floating sections were incubated overnight with a primary rabbit anti-tyrosine hydroxylase antibody (1:200; Merck Millipore, AB152) to evaluate the viral expression cells. Subsequently, the samples were incubated with the secondary antibody, Alexa Fluor 488-conjugated cross-adsorbed goat anti-rabbit IgG (H + L) (1:100; Thermo Fisher Scientific, A11008). Fluorescent images of TH and ChrimsonR-fused tdTomato were acquired using a fluorescence microscope (BZ-X800L/BZ-X810, BZ-H4XD multistack module and BZ-H4XF sectioning module [Keyence Co., Osaka, Japan]). The acquired images were processed and analyzed using ImageJ or Adobe Photoshop software.

### Quantification and statistical analysis

#### Statistics

In the lesion experiments, differences in the correct response and rule learning rates between the saline-infused and 6-OHDA-infused VTA groups were assessed using a two-way mixed ANOVA. In the pharmacological experiments, differences in the number of laps between the saline-infused and SCH23390-infused groups were assessed using a two-way repeated measures ANOVA and differences in the correct response and rule learning rates between the wild-type and saline-infused groups were assessed using Wilcoxon rank-sum test. In the optogenetic experiments, the control group without light stimulation (control-stim) was compared with the groups with pre-stimulation, post-stimulation, and opposite-stimulation using a two-way repeated-measures ANOVA. Both two-way mixed ANOVA and two-way repeated-measures ANOVA were performed using the ranova function in MATLAB. In the concurrent optogenetic and fiber photometry experiments, single or multiple Wilcoxon signed-rank tests with Bonferroni corrections were performed using the sign-rank function in MATLAB.

The shuffling distribution of the correct and rule learning rates was calculated by randomly shuffling the trial number 1000 times. We confirmed that the mice learned each task if the correct rate or rule learning rate exceeded the entire distribution (p < 0.001).
