# Supplementary material for: Optogenetic activation of the ventral tegmental area-hippocampal pathway facilitates rapid adaptation to changes in spatial goals
Source: iScience. 2023 Nov 23;26(12):108536. doi: 10.1016/j.isci.2023.108536 (PMC10711478; doi:10.1016/j.isci.2023.108536)
Supplement: Document S1. Figure S1 [file mmc1.pdf]

iScience, Volume *26*

Supplemental information

Optogenetic activation of the ventral tegmental area-hippocampal pathway facilitates rapid adaptation to changes in spatial goals

Yuta Tamatsu, Hirotsugu Azechi, Riku Takahashi, Fumiya Sawatani, Kaoru Ide, Fumino Fujiyama, and Susumu Takahashi

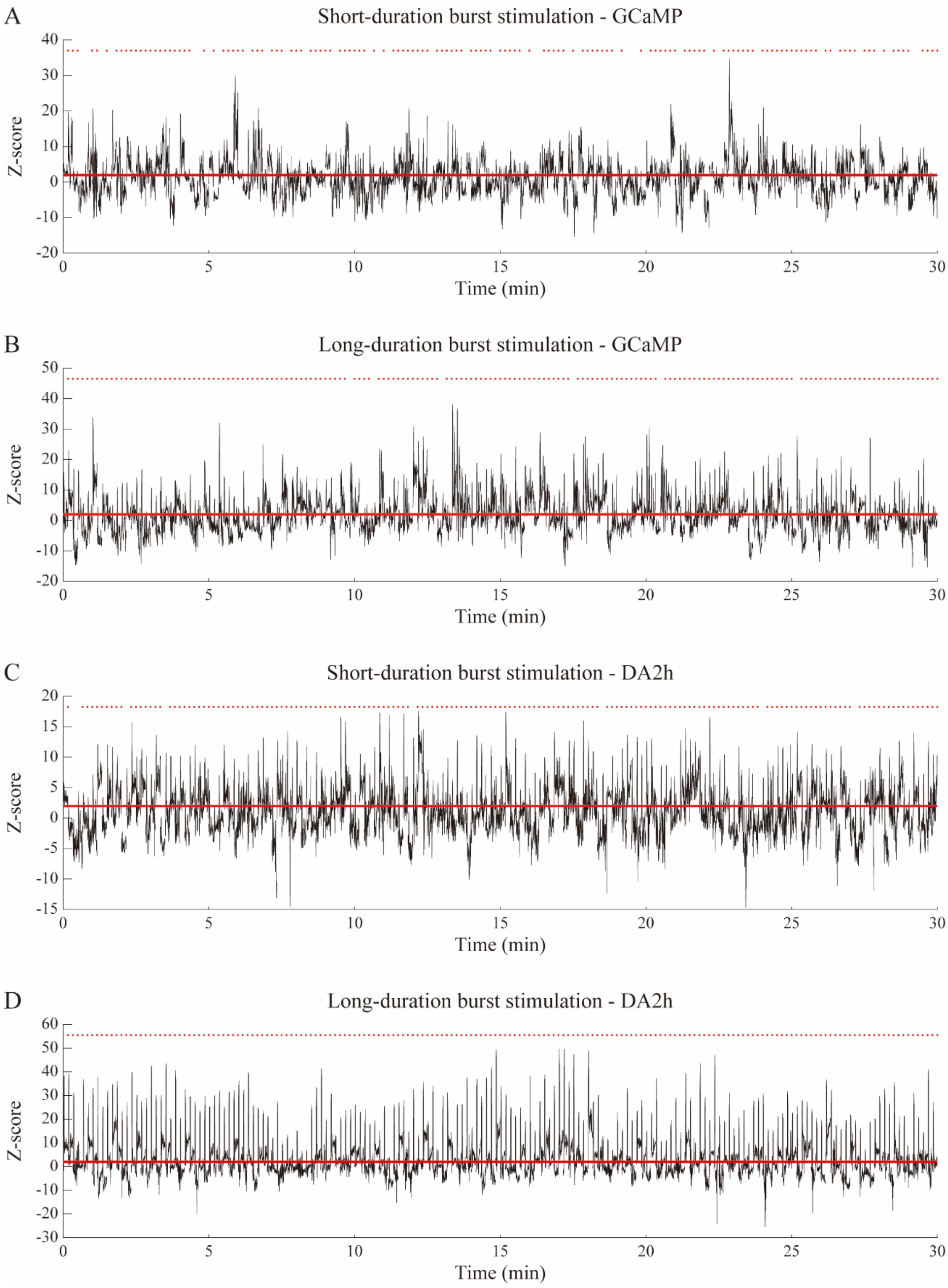

Figure S1. Calcium and dopaminergic responses in mice to burst stimulations, related to Figure 5.

(A-B) Representative normalized calcium signals of a mouse, expressed as z-scores, during burst stimulations of short (A) and long (B) durations are displayed. (C-D) Representative normalized dopaminergic responses of a mouse, expressed as z-scores, during burst stimulations of short (C) and long (D) durations are displayed. Red dots indicate responses to burst stimulation that exceed the predetermined threshold level, represented by the red line.
